# Planned delivery or expectant management for late preterm pre-eclampsia: study protocol for a randomised controlled trial (PHOENIX trial)

**DOI:** 10.1186/s13063-018-3150-1

**Published:** 2019-01-28

**Authors:** Lucy C. Chappell, Marcus Green, Neil Marlow, Jane Sandall, Rachael Hunter, Stephen Robson, Ursula Bowler, Virginia Chiocchia, Pollyanna Hardy, Edmund Juszczak, Louise Linsell, Anna Placzek, Peter Brocklehurst, Andrew Shennan

**Affiliations:** 1 King’s College London, London, UK; 2 Action on Pre-eclampsia, London, UK; 3 University College London, London, UK; 4 Newcastle University, London, UK; 5 National Perinatal Epidemiology Unit Clinical Trials Unit, University of Oxford, Oxford, UK; 6 Birmingham Clinical Trials Unit, University of Birmingham, Birmingham, UK

**Keywords:** Pre-eclampsia, hypertension, pregnancy, perinatal

## Abstract

**Background:**

Pre-eclampsia is a pregnancy disorder, characterised by hypertension and multisystem complications in the mother. The adverse outcomes of pre-eclampsia include severe hypertension, stroke, renal and hepatic injury, haemorrhage, fetal growth restriction and even death. The optimal time to instigate delivery to prevent morbidity when pre-eclampsia occurs between 34 and 37 weeks’ gestation, without increasing problems related to infant immaturity or complications, remains unclear.

**Methods/design:**

The PHOENIX trial is a non-masked, randomised controlled trial, comparing planned early delivery (with initiation of delivery within 48 h of randomisation) with usual care (expectant management) in women with pre-eclampsia between 34^+ 0^ and 36^+ 6^ weeks’ gestation. The primary objectives of the trial are to determine if planned delivery reduces adverse maternal outcomes, without increasing the short-term harm to infants (composite of perinatal deaths or neonatal unit admissions up to infant hospital discharge) or impacting long-term infant neurodevelopmental status at 2 years corrected age (Parent Report of Cognitive Abilities-Revised).

**Discussion:**

Current practice in the UK at the time of trial commencement for management of pre-eclampsia varies by gestation. Previous trials have shown that in women with pre-eclampsia after 37 weeks of gestion, delivery is initiated, as maternal complications are reduced without increasing fetal risks. Prior to 34 weeks of gestation, usual management aims to prolong pregnancy for fetal benefit, unless severe complications occur, necessitating preterm delivery. This trial aims to address the uncertainty for women where the balance of benefits and risks of delivery compared to expectant management are uncertain. Previous trials in this area have been undertaken, but have not provided a definitive answer, and the research question remains active. The results of this trial are expected to influence clinical practice internationally, through direct adoption and by incorporation into guidelines in countries with similar settings.

**Trial registration:**

ISRCTN01879376. Registered on 25 November 2013.

**Electronic supplementary material:**

The online version of this article (10.1186/s13063-018-3150-1) contains supplementary material, which is available to authorized users.

## Background

In the UK, 10–15% of pregnant women develop hypertension in pregnancy, and 2–5% pre-eclampsia. Pre-eclampsia is a multisystem disorder, characterised by placental and maternal vascular dysfunction, which is associated with substantial morbidity and mortality for the mother and infant. Adverse outcomes of pre-eclampsia include severe hypertension, stroke, renal and hepatic injury, haemorrhage, fetal growth restriction and even death [1].

Timely delivery may be indicated to prevent maternal and infant morbidity. Standard management of pre-eclampsia involves close monitoring whilst taking into consideration gestational age, fetal well-being and progression of maternal disease. When a diagnosis of pre-eclampsia is made at or beyond 37 weeks’ gestation, it is currently recommended that delivery be induced, since maternal and fetal risks can be significantly reduced without any apparent added risk associated with the intervention.

Around half (40%) of all pre-eclampsia occurs preterm (before 37 weeks), and these cases have the most serious outcomes. Using data from previous pre-eclampsia trials [2, 3], we have estimated that 33% of women with pre-eclampsia will present between 34^+ 0^ and 36^+ 6^ weeks of gestation and not require immediate delivery. Delivery before 34 weeks (meta-analysis of two randomised controlled trials, *n* = 133) [4] increases neonatal risk (Hyaline Membrane Disease risk ratio 2.3 [95% confidence interval, CI 1.39 to 3.81] and necrotising enterocolitis risk ratio 5.54 [95% CI 1.04 to 29.56]) without sufficient benefit in maternal outcomes.

The optimal time to instigate delivery to prevent morbidity when pre-eclampsia occurs between 34 and 37 weeks’ gestation, without increasing problems related to infant immaturity or complications, remains unclear. Current management involves close surveillance and delivery only when evidence of impending serious morbidity becomes apparent (e.g. deteriorating maternal or fetal condition). As this may be rapid or unexpected, planned delivery (the proposed intervention) beyond 34 weeks may be valuable. Neonatal and infant mortality and morbidity (e.g. through respiratory distress syndrome) may, nonetheless, occur following delivery between 34 and 37 weeks of gestation. However, neurodevelopmental morbidity and risk of growth restriction and death may be reduced by early delivery, and adverse events related to expectant management (including placental abruption, stillbirth and worsening growth restriction) may be decreased.

It is highly likely that routine delivery will reduce the maternal risk, as delivery cures pre-eclampsia. There is, therefore, a need to compare a policy of planned delivery between 34^+ 0^ and 36^+ 6^ weeks of gestation with that of expectant management, to evaluate the benefits and risks for the mother and baby, including assessment of longer-term neurodevelopmental outcomes.

This aim of this study is to compare planned delivery with expectant management (usual care) in women with pre-eclampsia between 34 and 37 weeks’ gestation. This study arose from a commissioned call by the funder (National Institute for Health Research), following development of the proposal and choice of important outcomes by clinicians with patient and public involvement. The study will be conducted according to the principles of the Declaration of Helsinki (dated 2008) and all applicable regulatory requirements. This protocol will be submitted to an NHS research ethics committee (REC) and the NHS Trust Research and Development Offices for approval.

## Methods/design

### Trial objectives

The aim of this study is to determine whether planned delivery in women with pre-eclampsia between 34^+ 0^ and 36^+ 6^ weeks’ gestation reduces maternal adverse outcomes without substantial worsening of neonatal or infant outcomes, compared with the current practice of expectant management to 37 weeks’ gestation.

#### Primary objectives

The primary objectives of the study are:

- To determine if planned early delivery for women with pre-eclampsia between 34^+ 0^ and 36^+ 6^ weeks’ gestation reduces adverse maternal outcomes based on a composite listed in the fullPIERS [5] paper with addition of recorded systolic hypertension (systolic blood pressure of ≥160 mmHg), as highlighted in the triennial Confidential Enquiry into Maternal Deaths (2006–8) [6].
- To evaluate the impact of the intervention on short- and long-term perinatal outcomes. Short-term outcomes will be assessed by a composite of perinatal deaths (antenatal or intrapartum stillbirths or neonatal deaths within 7 days, but not deaths due to congenital anomaly) or neonatal unit admissions up to time of infant hospital discharge.
- To determine the impact on infant neurodevelopmental status at 2 years of age corrected for prematurity using the Parent Report of Cognitive Abilities, Revised (PARCA-R) [7] Composite.

#### Secondary objectives

The secondary objectives of the study are:

- To investigate the effect of the intervention on other secondary maternal and neonatal short-term outcomes.
- To assess the impact of both management strategies on health-care resource use and quality-adjusted life years (QALYs)
- To assess the impact of both management strategies on health economic outcomes
- To evaluate quality of life using questionnaires immediately after randomisation and at 6 months and 2 years corrected age.

### Trial design

This will be a pragmatic, multicentre, randomised controlled trial of planned delivery versus expectant management in 900 women with pre-eclampsia between 34^+ 0^ and 36^+ 6^ weeks of gestation inclusive. The trial will be conducted in approximately 35 to 45 consultant-led maternity units across England and Wales. An internal pilot phase will initially be run, involving six centres over a period of 6 months to establish whether the procedures and assessments are conducive to achieving the recruitment and other objectives of the study. Recruitment is anticipated to take 36 months based on an assumption that each centre will on average recruit 1.5 women per month, with some allowance for unforeseen events and centres recruiting slower than expected. The study, including set-up, pilot phase, completion of mother and infant follow-up and reporting, is anticipated to take 72 months to complete. If the processes are shown to work in the internal pilot phase, recruitment to the main trial will proceed with no break and data from the internal pilot phase will be analysed together with the main trial data collected. The internal pilot will aim to recruit a total of 41 women by the end of month 6. Progression criteria (internal pilot into main trial) will be recruitment in the pilot study of at least 75% of target (31 women or more) with loss to follow-up of no more than seven women.

### Selection and withdrawal of participants

#### Inclusion criteria

Women who meet the following criteria will be eligible for enrolment into the study:

- Pregnancy of between 34^+ 0^ and 36^+ 6^ weeks of gestation inclusive
- Pre-eclampsia defined by the International Society for the Study of Hypertension in Pregnancy 2014 [8] as (1) diastolic blood pressure ≥90 mmHg (twice, ≥4 h and <1 week apart) or (2) diastolic blood pressure ≥110 mmHg on one occasion [9] and one or more of the following: (i) proteinuria (≥0.3 g/day by 24-h urine collection or ≥ 30 mg/mmol by spot urinary protein creatinine ratio), (ii) thrombocytopenia (platelet count < 150 × 10^9^/L), (iii) renal insufficiency (creatinine ≥90 μmol/L), (iv) impaired liver function (alanine transaminase or aspartate transaminase >70 IU/L), (v) fetal growth restriction (Estimated fetal weight EFW <10th centile confirmed by ultrasound); or superimposed pre-eclampsia
- Singleton or dichorionic diamniotic twin pregnancy
- Viable fetus
- Aged 18 years or over at the time of screening
- Able to give written informed consent

Women with any other co-morbidity (including pre-existing hypertension, diabetes etc.) or having had a previous caesarean section or with the fetus in any position will be eligible.

#### Exclusion criterion

Women will be excluded from participation in the study if a decision has already been made to deliver within the next 48 h.

### Recruitment, eligibility and consent

Members of the research team will provide a full verbal explanation and written description of the trial to women who meet the inclusion criteria (as above). The woman will be given sufficient time to consider the information, and to decide whether she will participate in the trial. Written informed consent will be sought from the woman and taken by an appropriately trained doctor.

### Study periods

A woman’s participation in the study may be from 34 weeks’ gestation until their child reaches 2 years of age corrected for prematurity, a maximum duration of 28 months.

### Withdrawal of participants

Women will be able to withdraw their consent at any time without giving a reason. Withdrawal from the study will not affect their (or their baby’s) ongoing care and there will be no requirement for any study-related follow-up safety assessments. The intervention may be discontinued if their clinician feels it is in the baby’s best interests. For a woman allocated to the expectant management group, the attending clinician will make a decision for delivery based on the National Institute for Health and Care Excellence (NICE) guidelines, with delivery planned at 37 weeks’ gestation. If clinical needs dictate delivery prior to 37 weeks’ gestation, this will not constitute withdrawal from the trial allocation. For a woman allocated to the planned delivery group, if the woman should decide that she does not wish to proceed with the planned delivery and instead chooses to continue to be monitored by her attending clinician, this will not constitute withdrawal from the study. There is no requirement to replace women who do not complete the study or need to be delivered prior to their planned delivery date.

### Assessment of outcomes

Outcomes will be recorded on the web-based database after a review of case notes by trained researchers after the discharge of the mother and baby. Confirmation of maternal outcome data (to include occurrence or not of the primary outcome) will be undertaken with an additional sign-off by the site’s principal investigator for each participant.

### Co-primary outcomes

#### Primary short-term maternal outcome

- Composite of maternal morbidity of fullPIERS [5] outcomes with the addition of recorded systolic blood pressure ≥160 mmHg (with or without medication) post-randomisation.

#### Primary short-term perinatal outcome

- Composite of perinatal deaths (antenatal or intrapartum stillbirths or deaths within 7 days of delivery but not deaths due to congenital anomalies) or neonatal unit admissions (physical separation of baby from the mother) prior to infant hospital discharge.

#### Primary long-term infant outcome

- PARCA-R composite score for neurodevelopment at 2 years of age corrected for prematurity [7].

### Secondary outcomes

Secondary maternal outcomes will include assessments of:

- Individual components of the composite primary outcome, as number of women with one or more of the following: eclampsia, Glasgow coma scale <13, stroke, hypertensive encephalopathy, posterior reversible encephalopathy, cortical blindness, retinal detachment, myocardial infarction, intubation other than for a caesarean section, pulmonary oedema, ionotropic support, saturation <92%, 50% FiO2 for > 1 h, infusion of a third parenteral antihypertensive, platelets <50 × 10^9^/L, disseminated intravascular coagulation, thrombotic thrombocytopenic purpura, haemolytic uraemic syndrome, acute fatty liver of pregnancy, hepatic dysfunction, haematoma or rupture, severe acute kidney injury (creatinine > 150 μmol/L or > 200 μmol/L in chronic kidney disease, dialysis, transfusion of blood products)
- Severe hypertension post-randomisation (systolic blood pressure ≥160 mmHg with or without medication on at least one occasion) recorded in written or electronic notes and measured by health-care professionals in clinical care
- Use of anti-hypertensive drugs recorded in written or electronic notes prescribed by health-care professionals in clinical care
- Progression to severe pre-eclampsia, defined as systolic blood pressure ≥160 mmHg, platelet count < 100 × 10^9^/L and abnormal liver function enzymes (ALT or AST > 70 IU/L)
- Estimated fetal weight (on ultrasound scan) <10th centile post-enrolment
- Absent or reversed end diastolic flow (on umbilical artery Doppler)
- Time and mode of onset (spontaneous, induced or pre-labour caesarean section) and mode of delivery (spontaneous vaginal delivery, assisted vaginal delivery or caesarean section)
- Confirmed thromboembolic disease requiring anticoagulation up to post-natal discharge
- Confirmed sepsis (positive blood or urine cultures) up to post-natal discharge
- Primary and additional indications for delivery in the expectant management arm: maternal hypertension not controlled by maximal therapy, biochemical abnormality, haematological abnormality, fetal compromise on ultrasound scan, fetal compromise on cardiotocography, severe maternal symptoms, 37 weeks of gestation or specified other
- Placental abruption

Secondary perinatal outcomes will include assessments of:

- Stillbirth post-randomisation
- Neonatal death prior to hospital discharge
- Admissions to neonatal unit
- Number of nights in each category of care (intensive, high dependency, special, transitional and normal)
- Total number of nights in hospital
- Birth weight (g)
- Customised/population birth weight centile
- Birth weight <10th and <3rd customised/population centile
- Gestational age at delivery
- Apgar score at 5 min post-birth
- Umbilical arterial and venous pH (and base excess) at birth
- Need for supplementary oxygen prior to discharge
- Number of days when supplemental oxygen is required
- Need for ventilation support (continuous positive airway pressure, high flow or endotracheal ventilation)
- Abnormal cerebral ultrasound scan
- Confirmed sepsis (positive blood or cerebrospinal fluid cultures)
- Necrotising enterocolitis (Bell’s stage 2 and 3)
- Seizures (confirmed by electroencephalography or requiring anticonvulsant therapy)
- Encephalopathy grade (worst at any time: mild, moderate or severe)
- Hypoglycaemia (blood glucose < 2.6 mmol/L on two or more occasions)
- Other indications and main diagnoses resulting in neonatal unit admission
- Exclusively breastfed at discharge from the neonatal unit

Secondary long-term maternal outcomes will include assessments of:

- Quality of maternal physical and mental health using the validated SF-12v2® questionnaire when the infant is 6 months and 2 years of age corrected for prematurity.

Secondary health economic and quality of life outcomes will include assessments of

- Quality of life using the validated quality of life questionnaire EQ-5D-5 L [10] immediately after randomisation, at 6 months and when the infant is 2 years of age corrected for prematurity
- Hospital attendances, nights and diagnostic tests from randomisation until delivery
- Cost of delivery
- Cost of neonatal care (hospital admissions, surgery and diagnostic tests)
- Retrospective 6-month health and social care use by mother and infant at 6 months and 2 years
- EQ-5D-5 L [10] for the calculation of maternal QALYs

### Trial procedures

**Fig. 1:**
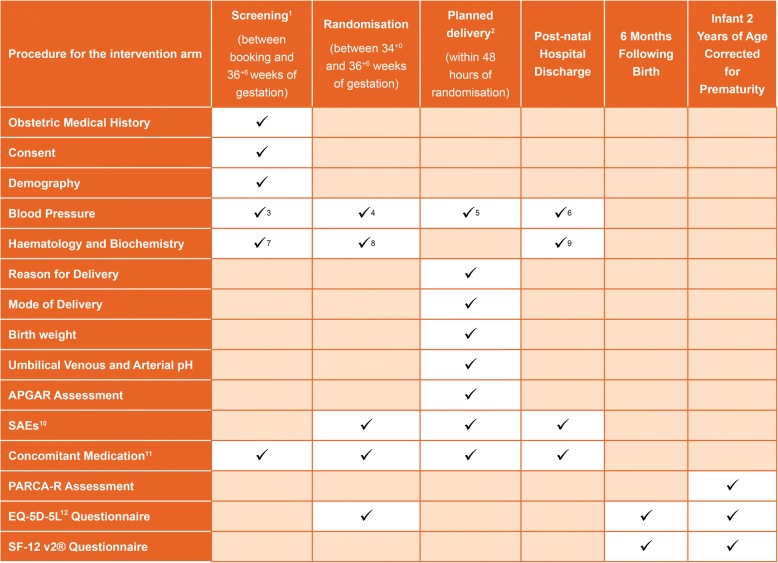
Schedule of participant enrolment, interventions and assessments in the trial. PARCA-R Parent Report of Cognitive Abilities, Revised, SAE serious adverse event. 1 Screening to be conducted of all women suspected of being eligible for the study. 2 Delivery to be commenced within 48 hours of randomisation for women randomised to the planned delivery group. 3 Eligibility for study to be assessed from blood pressure recorded at the time the diagnosis of pre-eclampsia. 4 Blood systolic pressure reading within the 48 hours prior to randomisation to be recorded. 5 Highest systolic blood pressure recorded between randomisation and delivery to be recorded. 6 Highest systolic blood pressure recorded between delivery and discharge to be recorded. 7 Haematology and/or Biochemistry results that contributed to diagnosis of pre-eclampsia to be recorded. 8 The most recent Haematology and/or Biochemistry results prior to randomisation to be recorded. 9 Abnormal Haematology/ Biochemistry results from randomisation to discharge to be recorded at discharge. 10 Serious Adverse Events (SAEs) to be recorded from randomisation to post-natal discharge. Only unexpected SAEs to be reported. 11 Brief details of anti-hypertensive and medication for induction will be recorded; all other concomitant medication will only be recorded in the event that an unexpected Serious Adverse Event is reported. 12 EQ-5D-5L[10] to be given to the participant to complete immediately after randomisation

The study procedures are shown in Fig. 1.

### Informed consent

Written consent will be sought from the woman only after she has been given a full verbal explanation and written description of the trial (via the participant information leaflet; Additional file 1). Women who do not speak English will be approached only if an adult interpreter is available. Relatives may not interpret. Introductory verbal and written information should be offered to all potentially eligible women with pre-eclampsia at the study’s recruiting centres. Written informed consent will be given using an informed consent form (Additional file 2) completed, signed and dated by the woman (with a countersignature by an interpreter where required) and signed by the person who obtained informed consent, who will be the principal investigator or another study doctor with delegated authority. After written informed consent has been obtained, a member of the research team will enter the baseline data onto the online database and perform randomisation, communicating the results directly to the woman. At all stages, it will be made clear to the woman that she is free to withdraw from the trial at any time without the need to provide any reason or explanation. Participants will be made aware that this decision will have no impact on any aspect of their continuing care.

The management of pregnant women whilst in hospital should be in accordance with the NICE guidelines for the management of hypertension in pregnancy [11]. Delivery will be in accordance with standard procedures but will most likely be through induction with prostaglandins, unless contraindicated. All options should be discussed with the pregnant woman and her needs and preferences taken into account.

Otherwise, women will be managed as follows.

### Intervention (planned delivery) group

The intervention is planned delivery with minimal delay (with initiation of delivery within 48 h of randomisation to allow for steroid use and neonatal cot availability). Use of corticosteroids will be at the discretion of the individual clinician as indicated in the NICE guidelines. Postnatal care should follow NICE guidelines [11, 12].

### Control (expectant management) group

Usual care is expectant management of pregnancy, as indicated by NICE guidelines and delivery at 37 weeks of gestation or sooner as clinical needs dictate. The NICE guidelines cover care on admission to hospital, treatment, measurement of blood pressure, testing for proteinuria and other parameters depending on whether the woman has mild or moderate hypertension.

If the woman has mild hypertension (blood pressure 140/90 to 149/99 mmHg), care would be as follows:

- Admission to hospital
- Measure blood pressure at least 4× a day
- No treatment of blood pressure
- No repeat quantification of proteinuria
- Blood test monitoring twice a week to determine kidney function, electrolytes, full blood count, transaminases and bilirubin.

If the woman has moderate hypertension (blood pressure 150/100 to 159/109 mmHg), care would be as for mild hypertension with the addition of the following assessments:

- Administration of oral labetalol to keep diastolic blood pressure between 80 and 100 mmHg and systolic blood pressure < 150 mmHg
- Blood test monitoring thrice a week to determine kidney function, electrolytes, full blood count, transaminases and bilirubin.

### Time of delivery: adherence to protocol

Following randomisation to either the planned delivery group (intervention) or expectant management group (control), the time of the onset of planned delivery (first method for induction of labour or time of planned caesarean section along with the indication) or onset of spontaneous labour will be recorded for all women. This will enable the monitoring of adherence to the protocol for both study groups so that protocol deviations can be identified and investigated.

### Sample size

The sample size for the PHOENIX study is calculated on the ability to observe a clinically significant risk reduction in the primary short-term maternal composite outcome of maternal morbidity and recorded systolic blood pressure of ≥160 mmHg, measured after randomisation. It is also powered to ensure that a clinically relevant effect size can be detected for the short- and long-term perinatal co-primary outcomes.

#### Superiority hypothesis for the primary short-term maternal outcome

Based on data from the PELICAN study [2], 49 of 115 women with suspected pre-eclampsia (42.6%, 95% CI 33.4% to 52.2%) enrolled between 34^+ 0^ and 36^+ 6^ weeks of gestation developed maternal morbidity and systolic hypertension of ≥160 mmHg. Therefore, assuming an expected adverse maternal outcome incidence of 43% in the control group (expectant management), a sample size of 850 women will be needed to demonstrate a relative risk reduction of 25% to 32.25% (deemed clinically significant) with a two-sided 5% significance level and 90% power in the planned delivery group. Taking into account a 5% loss of women in the follow-up, the overall target sample size for the study is 900 women (450 per group).

#### Non-inferiority hypothesis for the neonatal outcomes

A sample size of 850 women will result in approximately 860 live births (assuming 1 in 80 pregnancies are twin pregnancies). The PELICAN study [2] reported that a composite of perinatal death or any neonatal admission occurred in 27 of 115 infants (23.5%, 95% CI 16.1% to 32.3%). Assuming a composite adverse neonatal outcome incidence of 24% in the control group (expectant management), a sample size of 860 (430 per group) will achieve 93% power to detect a non-inferiority margin of a difference in incidence of no less than 10% (judged to be clinically relevant) and 78% power to detect a margin of no less than 8%.

To examine the component of perinatal death specifically, using Office for National Statistics data for all babies born in England and Wales in 2013, it is estimated that 1.6% (585/36,939) of all preterm births were perinatal deaths (stillbirth and early neonatal) [13]. A similar incidence is expected in women with pre-eclampsia as those deaths prevented by increased surveillance would be offset by pre-eclampsia associated co-morbidities of fetal growth restriction and placenta abruption. Thus, for the component of perinatal death, assuming a control group incidence of 1.5%, a sample size of 430 in each group would achieve 90% power to detect a non-inferiority margin of a difference in incidence of no less than 2.7%. For the component of neonatal unit admission, assuming a control group incidence of 21%, a sample size of 430 in each group will achieve 90% power to detect a non-inferiority margin of a difference in incidence of no less than 9%.

Assuming a loss to follow-up at 2 years of 20%, we should obtain long-term outcomes for approximately 690 infants (345 per group assuming no difference in the loss to follow-up between the groups). The PARCA-R questionnaire [7] provides a composite score for neurodevelopment with a standardised mean of 100 and standard deviation of 15. With a one-sided significance level of 2.5%, under a non-inferiority hypothesis, a sample size of 345 in each group achieves 94% power to detect a non-inferiority margin of a difference in the mean PARCA-R score of no less than 4 points (1/4 of a standard deviation). A margin of no less than 3 points can be detected with 75% power. Considering the results from the INFANT trial [14] of over 46,000 higher risk women with babies of over 35 weeks’ gestation, the original power calculation was revised. Using a revised standard deviation of 34, a sample size of 345 in each group will detect a non-inferiority margin of a difference in the mean PARCA-R score of no less than 9 points (1/4 of a standard deviation) with 94% power. A margin of no less than 7 points can be detected with 77% power.

In summary, a sample size of 900 women will have at least 90% power to detect: (1) a relative risk reduction of 25% (from 43% to 32%) in the primary maternal composite outcome, (2) a non-inferiority margin of a difference in the incidence of the primary short-term perinatal outcome of no less than 10% (from 24%) and (3) a non-inferiority margin of a difference in the mean 2-year PARCA-R score of ≥9 points.

### Randomisation

The allocation ratio for the intervention (planned delivery) to usual care (expectant management) will be 1:1. Randomisation will be managed via a secure web-based randomisation facility hosted by MedSciNet with a telephone back-up available at all times. A minimisation algorithm will be used to ensure balance between the groups with respect to the collaborating hospital, singleton or twin pregnancies, severity of hypertension in the 48 h prior to enrolment (highest systolic blood pressure with or without medication: ≤149 mmHg, 150–159 mmHg, ≥160 mmHg), parity (delivery of a baby past 24 weeks' gestation), previous caesarean sections and gestational age at randomisation (34, 35 or 36 weeks). MedSciNet will write the randomisation program and hold the allocation code. Following randomisation, the obstetrician will then arrange for delivery or ongoing expectant management as indicated by the randomisation.

### Masking

Due to the nature of this study, masking of clinicians, nursing staff and participants is not possible.

### Data collection

#### Data collection before postnatal discharge

Much of the outcome data for this trial are routinely recorded clinical items that can be obtained from the clinical notes or local hospital results system. No additional blood or tissue samples are required for this study. Women will be asked to complete the EQ-5D-5 L [10] questionnaire at the time of randomisation, which usually takes fewer than 5 minutes.

#### Data collection after discharge

Questionnaires will be sent to all participants at 6 months post-delivery and 2 years of age corrected for prematurity. The 6-month and 2-year questionnaires will collect the following data: EQ-5D-5 L [10], SF-12v2® [15] and maternal and infant health and social care use after their hospital discharge. In addition, the PARCA-R questionnaire [7] will be collected at 2 years.

### Data processing

All hospital trial data will be collected using bespoke electronic case report forms and entered directly into the study’s electronic database by the centre’s research staff. Data will be stored by single-entry only and at the point of entry, the data will undergo a number of validation checks to verify the validity and completeness. An additional sign-off of the maternal outcomes data will be performed by the site’s principal investigator for each participant. Follow-up questionnaires returned by post or completed by the mother on-line will also undergo a number of validation checks at the point of entry.

### Assessment of safety

A Data Monitoring Committee (DMC) will be established to ensure the wellbeing of study participants. The DMC will periodically review study progress and outcomes as well as reports of unexpected serious adverse events (SAEs). The DMC will, if appropriate, make recommendations to the Trial Steering Committee (TSC) regarding continuance of the study or modification of the study protocol.

### Adverse events

An adverse event is any untoward medical occurrence in a participant. It does not necessarily have to have a causal relationship with this intervention. Due to the high incidence of adverse events routinely expected in this patient population (e.g. abnormal laboratory findings, new symptoms etc.), only those adverse events identified as serious will be recorded for the trial.

### Serious adverse events

An SAE is any untoward medical occurrence that:

- results in death
- is life-threatening
- requires participant hospitalisation or prolongation of existing hospitalisation
- results in persistent or a significant disability or incapacity
- is a congenital anomaly or birth defect
- is an important medical event

### Expected SAEs

Expected SAEs are those events that are expected in the patient population or as a result of the routine care or treatment of a patient. The following events are expected in women with pre-eclampsia and their infants and as such do not require reporting as SAEs.

#### Expected maternal SAEs

- hepatic dysfunction
- hepatic haematoma or rupture
- coma or impaired consciousness (Glasgow coma score <13)
- cortical blindness
- reversible ischaemic neurological deficit
- retinal detachment
- acute renal insufficiency or failure
- postpartum haemorrhage requiring transfusion or hysterectomy
- platelet count <50,000
- severe uncontrolled hypertension
- myocardial ischaemia or infarction
- severe breathing difficulty
- pulmonary oedema
- sepsis
- admission to hospital for pre-eclampsia (if managed as an outpatient)

Although it is known that maternal death and strokes can occur in women with pre-eclampsia, they should still be reported as an SAE.

#### Expected infant SAEs

- perinatal death (unless unexpected in this population)
- congenital anomaly
- low birth weight
- reversed end diastolic flow
- requirement for supplemental oxygen or ventilation support
- intraventricular haemorrhage
- sepsis confirmed by positive cerebrospinal fluid or blood cultures
- necrotising enterocolitis
- seizures
- encephalopathy
- hypoglycaemia

Although it is known that neonatal death and stillbirth can occur in infants born to women with pre-eclampsia, they should still be reported as an SAE.

### Unexpected SAEs

An unexpected SAE is any event that meets the definition of an SAE and is not detailed in the list above as expected. The following unexpected SAEs must be reported:

- maternal death
- maternal stroke
- stillbirth
- neonatal death

### Safety reporting procedures

#### SAE recording

All SAEs (as described above) will be recorded from randomisation to the post-natal discharge from hospital of mother and baby.

#### Unexpected SAE reporting

Only unexpected SAEs will be reported. These will be followed up until the post-natal discharge of mother and baby from acute hospital care. Unexpected SAEs for both the mother and infant will be recorded and reported to the Clinical Trials Unit of the National Perinatal Epidemiology Unit (NPEU) within 24 h of research staff at the site becoming aware of the event. An SAE occurring to a participant will be reported to the REC, which gave a favourable opinion of the study, if, in the opinion of the chief investigator, the event was related (resulted from the administration of any of the research procedures) and unexpected in relation to those procedures. Reports of related and unexpected SAEs will be submitted within 15 working days of the chief investigator becoming aware of the event, using the Health Research Authority’s form for reporting an SAE. All reported SAEs will be regularly reviewed by the DMC throughout the study. The chief investigator will inform all investigators concerned of relevant information that could adversely affect the safety of participants.

#### Direct access to source data and documents

Direct access to source data and documents (including hospital records and notes, clinical charts, laboratory reports, pharmacy records and test reports) will be granted to authorised representatives from the NPEU Clinical Trials Unit, the sponsor and host organisations to permit study-related monitoring, audits and inspections.

### Statistical analysis

The primary analysis for all maternal outcomes will be by intention to treat with participants analysed in the groups to which they are assigned regardless of protocol non-compliance. The primary analyses for all perinatal and infant outcomes will be by intention to treat and per protocol, since the hypothesis under examination for these outcomes is a non-inferiority hypothesis. The intention-to-treat analysis will include data from all women who discontinue the intervention for any reason and data from all women who withdraw, up to the point of their withdrawal (after which no further data will be collected). The per-protocol analysis will exclude babies of women who do not receive the allocated intervention as per protocol. Non-inferiority will be concluded if the results from both populations are consistent with each other. If women in the expectant management arm are delivered prior to 37 weeks’ gestation due to clinical need (i.e. with new indications for delivery by NICE guidelines [11, 12]), this will not be considered a protocol deviation. A sensitivity analysis will also be carried out excluding babies of women randomised to the planned delivery arm where initiation of delivery is more than 96 h post-randomisation. This is to allow for clinical (e.g. steroid administration) and logistical (e.g. availability of labour ward bed or neonatal unit cot) delays.

All outcomes will be analysed adjusting for minimisation factors (as listed above) at randomisation [16] where possible. Binary outcomes will be analysed using log binomial regression models. If the model does not converge, log Poisson regression models with robust variance estimation will be used [17]. Results will be presented as adjusted risk ratios with associated CIs. The site will be treated as a random effect in the model, and all other factors as fixed effects. For continuous outcomes, differences in means and associated CIs will be estimated using linear regression models assuming the residuals are normally distributed. Should this assumption be considered unmet, quantile regression methods will be used. For all primary outcomes, 95% CIs will be presented. For the analysis of perinatal outcomes, the adjusted analysis will also account for the correlation of outcomes in twins included in the trial by treating these as random effects in the model.

The frequency and content of any interim analyses, including any stopping guidelines, will be determined by an independent DMC and documented in the DMC’s charter.

Further details of the short-term outcomes can be found in the statistical analysis plan (Additional file 3). A statistical analysis plan for the 2-year follow-up analysis is currently in development.

The loss to long-term follow-up is expected to be around 20% to 30% and to be associated with poor outcomes and lower socioeconomic status. Babies for whom no 2-year follow-up data are received will be compared to babies with 2-year data on demographic and clinical characteristics as well as short-term outcomes.

### Economic evaluation

An economic evaluation from the perspective of the National Health Service (NHS) and personal social services will be conducted alongside the main trial. Data on health and social care resource utilisation will be collected using patient administration systems, maternity and neonatal databases, and logs of tests and procedures, together with data from questionnaires administered at 6 months and 2 years, which capture health and social care resource use for mother and child in the previous 6 months. Health and social care services will be costed using national published sources (NHS reference costs and Unit Costs of Health and Social Care, Personal Social Services Research Unit, British National Formulary). QALYs for the mother will be calculated from EQ-5D-5 L [10] utility scores collected at baseline, 6 months and 2 years and the SF-12v2® [15] questionnaire also at 6 months and 2 years. Differences in infant mortality between the two groups will be captured by assuming full health up to 2 years for surviving infants. The final results of the economic evaluation analyses will be expressed as the mean incremental cost per mean QALY gained from baseline until the 2-year follow-up. Costs and QALYs in the second year will be discounted in line with NICE guidance [18]. As the analysis will be undertaken after the 2-year follow-up, a full health economic analysis plan, linking with the 2-year statistical analysis plan, is currently in development.

### End of trial

The PHOENIX trial has two phases: an intervention phase and a follow-up phase. The end of the intervention phase will be when the last participating mother and infant have been discharged from hospital. For regulatory purposes, the end of the study is defined as the date when the study database is locked. An end of study declaration will be made to the approving REC within 3 months of this date.

### Early cessation

Based on interim data on the trial’s outcomes, adverse event data, accumulating evidence from other trials and any other evidence from relevant studies, the DMC will inform the TSC if, in its view, there is proof beyond reasonable doubt that the data indicate that the trial should be terminated. This will apply if any part of the protocol under investigation is either clearly indicated or contra-indicated, either for all infants or for a particular subgroup of trial participants. A decision to inform the TSC of such a finding will, in part, be based on statistical considerations. Appropriate proof beyond reasonable doubt cannot be specified precisely. A difference of at least 3 standard errors in the interim analysis of a major outcome may be needed to justify halting or modifying the study prematurely, for the superiority hypothesis.

### Participant confidentiality, data handling and record keeping

Overall responsibility for ensuring that each participant’s information is kept confidential will lie with the study sponsor. All paper documents will be stored securely and kept in strict confidence in compliance with the Data Protection Act (1998) and the General Data Protection Regulation. Data entered onto electronic case report forms will be automatically transferred for storage in an electronic database held by MedSciNet on behalf of the sponsors. Participants will be identified only by a study-specific number and their initials. The participant’s name and any other identifying details will be stored in a separate database also held by MedSciNet on behalf of the sponsors. The databases will be linked only by the participant’s study number. This identifiable information will be collected and retained with the participant’s explicit consent to enable follow-ups to be undertaken. After the study has completed and the reports published, the data will be archived in a secure physical or electronic location with controlled access.

Electronic files will be stored on a file server that has restricted access. The server is in a secure location and access is restricted to a few named individuals. Authorisation to access restricted areas of the NPEU Clinical Trials Unit network is as described in the unit’s security policy. Data will be processed on a workstation by authorised staff. All paper documents will be stored securely and kept in strict confidence in compliance with the Data Protection Act (1998) and the General Data Protection Regulation and all trial data will be stored in line with the Medicines for Human Use (Clinical Trials) Amended Regulations 2006. Due to the nature of pregnancy research, data will be kept for a period of no fewer than 25 years to allow follow-ups on health-related issues that may become relevant. All personal data will be held securely at all times and will not be used for any other purpose.

The dataset will be available to appropriate academic parties on request from the chief investigator in accordance with the data-sharing policies of King’s College London and the NPEU Clinical Trials Unit, with input from the co-investigator group where applicable.

### Retention of personal data

Personal data will be used to contact the participant, to thank them for participating in the study, to facilitate the follow-ups at 6 months and 2 years of age, to co-ordinate the follow-ups and to disseminate the results of the study to participants.

### Data security

An IT security risk assessment of MedSciNet will be undertaken by the sponsor. Prior to recruitment commencing, data-sharing agreements will be produced to ensure that all study data are captured and stored as per the sponsor’s security policy and in compliance with all UK data storage requirements. A similar risk assessment and data-sharing agreement will also be produced to ensure EQ-5D-5 L data captured via the EuroQol website are captured, stored and transferred to the MedSciNet database as per the sponsor’s security policy.

### Quality control and assurance

#### Site initiation and training

The site principal investigator and a local research midwife or nurse, or their delegates, from each recruiting centre will be fully trained in the protocol and data collection procedures. They will then be responsible for delivering this training to all relevant site staff to make sure that they are conversant with the trial’s procedures prior to opening their centre for recruitment. The site research team will also promote the trial so that the necessary recruitment targets are reached within the timescale and they will encourage recruitment in their centre.

#### Site monitoring and auditing

The site research team will be responsible for the day-to-day smooth running of the trial at a recruiting site. The Clinical Trials Unit will monitor recruitment against targets, provide staff education and training, and monitor the completeness and quality of collected data. The study monitor will perform regular visits to all recruiting centres and will verify the source data for selected participants during these visits.

Throughout the trial, there will be central monitoring, overseen by the Project Management Group, DMC, TSC and Quality Assurance team to ensure there is good communication between the NPEU trial team and site staff. Trial monitoring will be conducted in accordance with the monitoring plan developed from the trial-specific risk assessment. The monitor will visit sites where anomalies are identified through central monitoring. Sites that are identified as requiring additional support will be visited by a member of the trial team or the monitor as appropriate to the particular issues.

The DMC will look regularly at protocol adherence by site and by trial arm, including randomisation processes and patterns of allocation.

### Risk assessment

A study risk assessment has been performed as part of the application to receive funding. This risk assessment will be reviewed at regular intervals during the study and will be updated as required.

### Communication

After REC approval has been obtained, this protocol will be submitted for publication and will be available for download via the NPEU website.

### Study website

The PHOENIX study website will provide information on the study to recruiting centres, participants and their families. Copies of all electronic case report forms, the study protocol, participant information leaflet and training literature will be available along with information on centres participating in the study and contact details for the coordinating centre. The page for participants will also have links to other websites that offer advice and support to people affected by pre-eclampsia.

## Discussion

Current practice in the UK at the time of trial commencement for management of pre-eclampsia varies by gestation. Previous trials have shown that if delivery is initiated after 37 weeks of gestation for women with pre-eclampsia, maternal complications are reduced without increasing fetal risks. Prior to 34 weeks of gestation, usual management is to aim to prolong pregnancy for fetal benefit, unless severe complications occur that necessitate a preterm delivery. This trial aims to address the uncertainty in the balance of benefits and risks of an earlier delivery compared to expectant management. Previous trials in this area have been undertaken, but have not provided a definitive answer, and the research question remains active. The results of this trial are highly likely to influence clinical practice internationally, through a direct impact and through impacting guidelines in countries with similar settings.

### Trial status

The current PHOENIX protocol is version 3.1 (5 January 2018). The trial opened to recruitment on 29 September 2014. The first participant was recruited on 29 September 2014. All 46 sites (40 NHS Trusts) were opened by 23 January 2018. Recruitment ended on 10 December 2018. Follow-up in progress until last woman and infant have been discharged from hospital for short-term outcomes and to 2 years post-delivery for long-term outcomes (Additional file 4).

## Additional files

Additional file 1: Participant information leaflet. (PDF 1587 kb)

Additional file 2: Consent form. (PDF 238 kb)

Additional file 3: Statistical analysis plan. (DOCX 187 kb)

Additional file 4: SPIRIT 2013 Checklist: Recommended items to address in a clinical trial protocol and related documents. (DOC 121 kb)

## Abbreviations

APEC: Action on pre-eclampsia
CI: Confidence interval
DMC: Data monitoring committee
NHS: National health service
NICE: National Institute for Health and Care Excellence
NPEU: National perinatal epidemiology unit
PARCA-R: Parent report of cognitive abilities, revised
QALY: Quality-adjusted life year
REC: Research ethics committee
SAE: Serious adverse event
TSC: Trial steering committee
