# Supplementary material for: Planned delivery or expectant management for late preterm pre-eclampsia: study protocol for a randomised controlled trial (PHOENIX trial)
Source: Trials. 2019 Jan 28;20:85. doi: 10.1186/s13063-018-3150-1 (PMC6350286; doi:10.1186/s13063-018-3150-1)
Supplement: Supplementary file 1 — Participant information leaflet. (PDF 1587 kb) [file 13063_2018_3150_MOESM1_ESM.pdf]

Phoenix

Pre-eclampsia in Hospital: Early Induction or Expectant Management

Participant Information Leaflet

Study title:

Pre-eclampsia in Hospital: Early Induction or Expectant Management

Short title:

Phoenix Study

Thank you for taking the time to read this information leaflet. We appreciate that it may be a difficult time to think about being involved in research but we feel it is important to tell you about a study that this hospital is taking part in. A member of the medical team will go through the information with you and answer any questions you may have.

REC Reference: 13/SC/0645

ISRCTN: 01879376

What is the purpose of this study?

Pre-eclampsia can be a serious condition for both mother and baby. Most women with pre-eclampsia make a full recovery and their babies are healthy. However, pre-eclampsia can cause a sudden, rapid rise in blood pressure and in severe cases can cause fits, strokes, multiple organ failure and very rarely, death. Babies whose mothers have pre-eclampsia tend to be smaller and are more likely to be born early. The cause of pre-eclampsia is not known.

Women with pre-eclampsia are usually admitted to hospital so they can be carefully monitored. Labour is normally started, by induction, between 37 and 38 weeks of pregnancy, by which time most babies are ready to be born. But, because some complications of pre-eclampsia can be life-threatening and the condition of both the mother and baby can suddenly worsen, some doctors think it may be better for women with pre-eclampsia to have their babies earlier than this.

This study aims to find out whether, in women with pre-eclampsia between 34 and 37 weeks of pregnancy, planned early birth (usually by induction), causes fewer complications for the mother and/or baby, compared to waiting until 37 weeks (unless a serious problem occurs before this time) . The study may help improve the care of women with pre-eclampsia in the future.

Why have I been invited to take part?

You are being invited to take part in the study because you have pre-eclampsia but your condition does not mean that your baby must be delivered immediately.

Approximately 900 women with pre-eclampsia, from approximately 35 hospitals in England and Wales, will be involved in the study which will take place over 3 years.

Do I have to take part?

No. It is entirely your decision whether to take part in the study or not. If you decide not to, your care and that of your baby will not be affected in any way. If you decide you would like to take part, you can change your mind at any time and withdraw from the study without having to give a reason.

What will happen if I do take part?

If you agree to take part, you will be asked to sign a consent form.

Details about you and your pregnancy will be put into a computer which will randomly choose whether your labour will be induced as soon as possible (in the Planned Delivery Group) or whether you will be monitored carefully in hospital and have your labour started by induction after 37 weeks of pregnancy, or sooner if a more serious problem arises (in the Expectant Management Group).

You will have a 50/50 chance of being in either group. Whichever group you are in, you will be given a questionnaire to complete about your health before your baby is born.

If you are in the **Planned Delivery Group**, your doctor will plan to start the induction of your labour within 48 hours. Your doctor may give you two steroid injections (12 or 24 hours apart) to help your baby's lungs mature before inducing you. For most women, your midwife will then give you a hormone called prostaglandin, this is usually a vaginal gel/pessary, to start labour. If you don't go into labour, your doctor will start the birth of your baby in other ways following your hospital's standard practice.

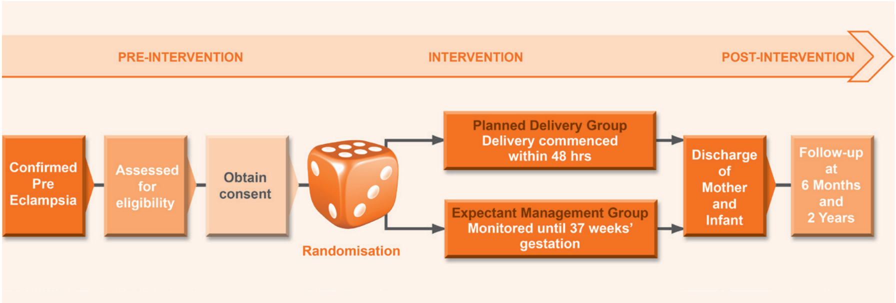

If you are selected to be in the **Expectant Management Group**, your doctors and midwives will carefully monitor you and your baby until you reach 37 weeks of pregnancy and then arrange for your labour to be induced in the same way as for women in the Planned Delivery Group. But, if the doctors/ midwives are worried about the health of either you or your baby before you reach 37 weeks of pregnancy, they may decide to deliver your baby earlier than this. You will still be included in the study if this happens.

After you have given birth, you and your baby will be cared for in the usual way at your hospital. It will not make a difference which group you were in.

Information (on your health and feelings) will be collected about you and your baby until you are both discharged from hospital.

Six months after the birth of your baby, you will be sent a similar questionnaire to the one you completed before your baby was born: this also records which NHS services you and your baby have used during the study. You will also be sent a questionnaire shortly after your baby reaches his/her second birthday (two years after their due date), along with a questionnaire about the health of your child at this age.

What are the possible risks and benefits of taking part?

There may be both risks and benefits in taking part which is why we feel it is important to do this study to improve care for women with pre-eclampsia.

If you in the Planned Delivery Group, your baby may be born up to 3 weeks (at 34 weeks of pregnancy) before full term (a baby at 37 weeks is considered at term, but most pregnancies go onto 40 weeks). This may mean your baby has problems associated with being born early. If you are in the Expectant Management Group, you and/or your baby may become more unwell from pre-eclampsia because you are pregnant for longer. When mothers have pre-eclampsia sometimes their babies do not get enough nutrients for them to grow properly and you could become very ill from the associated conditions of pre-eclampsia. This might mean you have to give birth quickly (either by induction or by Caesarean section), before 37 weeks of pregnancy, because your health or your baby's could get worse quickly, which may be upsetting.

We do not know which option is better for mother and/or baby, which is why we are doing the study.

Will taking part in this study be kept confidential?

If you take part in the study, some personal contact information will be collected. These details will be entered onto a secure online database and only members of the research team will use this information to contact you. Prior to us contacting you we will check the information you have given us, including NHS numbers, dates of birth, surnames and forenames, addresses and postcodes for you and your baby with The Health and Social Care Information Centre (a part of the NHS) . They will only provide us with updated information, such as a change to your address or health status. All information will be treated confidentially

No other information collected for the study will identify you or your baby.

Information will be on a separate secure online database designed specifically for this study and will only be accessible by those involved in the research. Unidentifiable data from this study may be shared with other researchers who are doing similar work.

To make sure that the study is being carried out correctly, your maternity records may be looked at by representatives of the study sponsors (Kings College London and Guy's and St Thomas' NHS Foundation Trust), study organisers and regulatory authorities.

What if relevant new information becomes available?

Sometimes we get new information about the treatment being studied. If this happens, any new information that is likely to affect your participation in the study in any way will be discussed with you. You will be free to decide whether to continue with the study. If you decide to withdraw, your care and that of your baby will not be affected.

If the study is stopped for any reason, we will tell you and let you know what will happen next.

What will happen if I don't want my baby and myself to continue with the study?

You can withdraw from the study at any time. We will ask you if we may use the data collected up to the point of your withdrawal in the analysis of the study.

What if there is a problem?

If at any stage you have any concerns about this study or the way it has been carried out, you should talk to the doctor or nurse who is leading the study in your hospital. You can also talk to the Patient Advice and Liaison Service (PALS) in your hospital. Their contact details are on the back cover of this leaflet.

If you remain unhappy and wish to complain formally, you can do this through the NHS Complaints Procedure. Details of this procedure can be obtained from the following website:

http://www.nhs.uk/choiceintheNHS/Rightsandpledges/complaints/Pages/NHScomplaints.aspx

In the unlikely event that something does go wrong and you or your baby is harmed during the research, then you may have grounds for legal action to obtain compensation against Kings College London and Guy's and St Thomas' NHS Foundation Trust who sponsor the study but you may have to pay your legal costs. The NHS Complaints Procedure (mentioned above) will still be available to you.

Involvement of your General Practitioner (GP)

With your permission, your GP will be informed of your participation in the study and they will be given a copy of this information leaflet for their records. They may then be able to help if you have queries about the study after you are discharged from hospital.

What will happen to the results of the research study?

At the end of the study, the results will be analysed and published in medical journals. We will send you a summary of the final results of the study. A copy of the full journal article can be requested from the Kings College London. You and your baby will not be identified in any report or publication about the study.

Who is organising and funding the research?

The study is the responsibility of the Kings College London and Guy's and St Thomas' NHS Foundation Trust but is run by the National Perinatal Epidemiology Unit, Clinical Trials Unit which is a department of Oxford University. It is funded by the Health Technologies Assessment programme within the National Institute for Health Research *[http://www.hta.ac.uk]*.

Your doctors and midwives will not be paid for enrolling you onto the study.

Who has reviewed the study?

All research that involves NHS patients has been approved by an NHS Research Ethics Committee before it goes ahead. Approval means that the Committee is satisfied that your rights will be respected, that any risks have been reduced to a minimum and balanced against possible benefits, and that you have been given sufficient information on which to make an informed decision to take part or not. The Hampshire B Research Ethics Committee has reviewed and approved this study.

Further information and contact details

Thank you for reading this leaflet – please discuss this study with the doctor or nurse who is looking after you if you have any questions.

You will be given a copy of this information sheet and signed consent form to keep.

Local contacts

Principal Investigator

Dr X
Hospital X
A Town

Tel: xxxx XXXXXXXX

Local Research Midwife

{_MIDWIVES_}
Hospital X
A Town

Tel: xxxx XXXXXXX

Patient advice and liaison services

{_PALS_}

Tel: xxxx XXXXXX Email: abcd@efghijklm.co.uk

Phoenix

Study Co-ordinating Centre

NPEU Clinical Trials Unit, Nuffield Department of Population Health
University of Oxford, Old Road Campus, Headington, Oxford, OX3 7LF

01865 289733

phoenix@npeu.ox.ac.uk

Information is also available on the study website
www.npeu.ox.ac.uk/phoenix

The PHOENIX study is funded by the National Institute for Health's HTA Programme. (project reference 12/25/03)

Guy's and St Thomas' NHS
NHS Foundation Trust
