# Supplementary material for: Planned delivery or expectant management for late preterm pre-eclampsia: study protocol for a randomised controlled trial (PHOENIX trial)
Source: Trials. 2019 Jan 28;20:85. doi: 10.1186/s13063-018-3150-1 (PMC6350286; doi:10.1186/s13063-018-3150-1)
Supplement: Supplementary file 2 — Consent form. (PDF 238 kb) [file 13063_2018_3150_MOESM2_ESM.pdf]

Phoenix
Pre-eclampsia in Hospital: Early Induction or Expectant Management

Consent Form

Please complete in black pen

Hospital name: ____________________ **Participant's Study Number:** ☐☐☐☐☐

Name of Principal Investigator: ____________________

		Please initial the boxes below
1.	I confirm that I have read and understood the Participant Information Leaflet dated 22 March 2016 (Version 4) for the above study. I have had the opportunity to consider the information, ask questions and have had these answered satisfactorily.	☐
2.	I understand that my participation is voluntary and that I am free to withdraw at any time without giving a reason and without my medical care or legal rights or that of my baby being affected.	☐
3.	I understand that relevant sections of the medical records and data collected during the study for my baby and I may be looked at by the study organisers, the research sponsors, or your NHS Trust. I give permission for these individuals to have access to our records where it is relevant to taking part in this research.	☐
4.	I agree that personal identifiable information will be collected and stored on the PHOENIX study database for use by the study's coordinating centre team to enable follow-up of myself and my baby and retained as explained in the participant information leaflet.	☐
5.	I understand that the information held and maintained by The Health and Social Care Information Centre and other central UK NHS bodies may be used to help contact me or provide information about my health status, and that of my baby. This is on the understanding that all information will be treated confidentially.	☐
6.	I agree to my GP being informed of my participation in the study.	☐
7.	I agree to take part in the study.	☐

Name of Participant	Name of doctor taking consent
Signature of Participant	Signature of doctor taking consent
D D / M M / Y Y	D D / M M / Y Y

PHOENIX Study Co-ordinating Centre

NPEU Clinical Trials Unit, National Perinatal Epidemiology Unit, University of Oxford, Old Road Campus, Headington, Oxford, OX3 7LF

T: 01865 289 733 F: 01865 289 740 E: phoenix@npeu.ox.ac.uk

www.npeu.ox.ac.uk/phoenix

The PHOENIX study is funded by the National Institute for Health's HTA Programme. (project reference 12/25/03)

King's College London · npeu Clinical Trials Unit · University of Oxford · Guy's and St Thomas' NHS Foundation Trust

Top copy to PHOENIX Co-ordinating Centre; one copy to participant, one copy to Site folder, and one copy to participants medical notes
