# Supplementary material for: Planned delivery or expectant management for late preterm pre-eclampsia: study protocol for a randomised controlled trial (PHOENIX trial)
Source: Trials. 2019 Jan 28;20:85. doi: 10.1186/s13063-018-3150-1 (PMC6350286; doi:10.1186/s13063-018-3150-1)
Supplement: Supplementary file 3 — Statistical analysis plan. (DOCX 187 kb) [file 13063_2018_3150_MOESM3_ESM.docx]

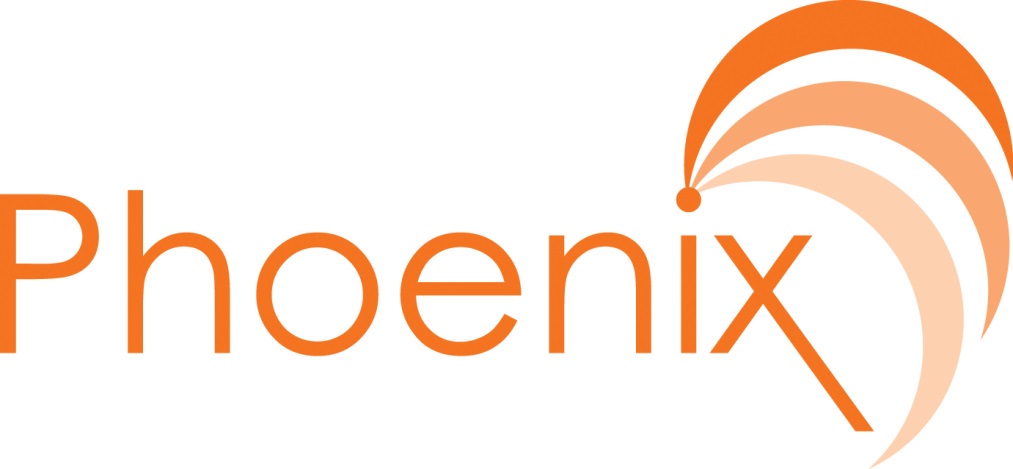


Full Title: Pre-eclampsia in Hospital: Early Induction or Expectant Management

ISRCTN01879376

REC reference: 13/SC/0645

Statistical Analysis Plan (SAP)

Version 1.1, 17^th^ July 2018

Authors: Melanie Greenland (PHOENIX Trial statistician, NPEU CTU)

Pollyanna Hardy (Senior statistician, NPEU CTU)

Reviewers: Professor Lucy Chappell (Co-Chief Investigator)

Ed Juszczak (NPEU CTU Director)

Virginia Chiocchia (Trial Statistician from May 2017, NPEU CTU)

Louise Linsell (Senior Trial Statistician from May 2017, NPEU CTU)

### Document history

| Version | Date | Edited by | Comments |
| --- | --- | --- | --- |
| 0.1 | 10/08/15 | MG | First draft version created August 2015. |
| 0.2 | 24/11/15 | PH | Reviewed and updated by senior statistician (PH) |
| 0.3 | 25/11/15 | PH | Taking account of comments by LC, and based on telephone call with PH and LC on 25/11/15 |
| 0.4 | 04/12/15 | PH | Updated incorporating comments from DMC at meeting held 03/12/15. |
| 0.5 | 12/01/16 to 14/01/16 | MG/PH | Updates made following CIG meeting on 10/12/15. |
| 0.6 | 14/01/2016 | PH | Tracked changes accepted |
| 0.7 | 17/07/2017 | LC/LL | Updates made during stats meeting on 17/07/2017 and track changes by LL accepted |
| 0.8 | 07/08/2017 | VC/LL | Updates made by VC and LL following further review |
| 0.9 | 21/09/2017 | VC/LL/LC | Updates made by VC and LL following further review |
| 0.10 | 08/01/2018 | VC/LC | Updates made by VC and LC following further review |
| 0.11 | 14/02/2018 | VC | Updates made following review by PH |
| 1.0 | 24/04/2018 | VC | First final version to sign-off |
| 1.1 | 16/07/2018 | VC | Confidence intervals for secondary outcomes changed from 99% to 95% to comply with NPEU guidelines |

### Contents

[1. Introduction 7](#_Toc519599193)

[2. Background 8](#_Toc519599194)

[3. Description of primary and secondary outcomes 8](#_Toc519599195)

[3.1 Primary outcome 8](#_Toc519599196)

[3.2 Secondary outcomes 8](#_Toc519599197)

[4. Sample size and power 9](#_Toc519599198)

[5. Random allocation 10](#_Toc519599199)

[6. Protocol non-compliances 10](#_Toc519599200)

[6.1 Major 10](#_Toc519599201)

[6.2 Minor 11](#_Toc519599202)

[7. Interim analyses 11](#_Toc519599203)

[8. Data collection schedule 11](#_Toc519599204)

[8.1 Before discharge 11](#_Toc519599205)

[9. Derivation of variables 12](#_Toc519599206)

[10. Participant groups for analysis 12](#_Toc519599207)

[10.1 Primary analysis strategies 12](#_Toc519599208)

[10.2 Post randomisation exclusions 12](#_Toc519599209)

[10.3 Descriptive analysis population 13](#_Toc519599210)

[10.4 Comparative analysis population 13](#_Toc519599211)

[10.5 Interim analysis population 13](#_Toc519599212)

[11. Descriptive analyses 13](#_Toc519599213)

[11.1 Representativeness of trial population and participant throughput 13](#_Toc519599214)

[11.2 Baseline comparability of randomised groups 14](#_Toc519599215)

[11.3 Loss to follow-up 14](#_Toc519599216)

[11.4 Description of adherence to intervention 14](#_Toc519599217)

[12. Comparative analysis 14](#_Toc519599218)

[13. Pre-specified subgroup analysis 15](#_Toc519599219)

[14. Sensitivity analysis 15](#_Toc519599220)

[15. Safety data analysis 15](#_Toc519599221)

[16. Statistical significance 16](#_Toc519599222)

[17. Procedure for accounting for missing data 16](#_Toc519599223)

[18. Procedure for reporting deviation(s) from the original statistical plan 16](#_Toc519599224)

[19. Statistical software employed 16](#_Toc519599225)

[20. Additional exploratory analysis 16](#_Toc519599226)

[21. Dummy tables 16](#_Toc519599227)

[22. References 16](#_Toc519599228)

[23. Approvals 17](#_Toc519599229)

### List of abbreviations

| AE | adverse event |
| --- | --- |
| ALT | alanine transaminase |
| AST | aspartate aminotransferase |
| BMI | body mass index |
| BP | blood pressure |
| cm | centimetre |
| CNS | central nervous system |
| CPAP | continuous positive airway pressure |
| CTU | clinical trials unit |
| DBP | diastolic blood pressure |
| EEG | electroencephalography |
| EFW | estimating fetal weight |
| FiO2 | fraction of inspired oxygen |
| g | gram |
| gest | gestation |
| GROW | gestation related optimal weight |
| HELLP | haemolysis, elevated liver enzymes, low platelet count |
| HTA | Health Technology Assessment |
| hr | hour |
| hrs | hours |
| IQR | interquartile range |
| ITT | intention to treat |
| IU | international units |
| IVH | intraventricular haemorrhage |
| kg | kilogram |
| L | litre |
| LMWH | low molecular weight heparin |
| m | metre |
| max | maximum |
| MD | mean difference |
| min | minimum |
| mg | milligrams |
| ml | millilitre |
| mmHg | millimetres of mercury |
| mol | mole |
| MoM | multiples of the median |
| NIHR | National Institute for Health Research |
| NNU | neonatal unit |
| PAPP-A | pregnancy associated plasma protein-A |
| PARCA-R | performance of the parents report of children’s abilities - revised |
| PE | point estimate |
| pg | pictogram |
| pH | power of hydrogen |
| PI | pulsatility index |
| PIERS | pre-eclampsia integrated estimate of risk |
| PIGF | placenta growth factor |
| PP | per protocol |
| PROM | premature rupture of membranes |
| RCT | randomised controlled trial |
| RDS | respiratory distress syndrome |
| RR | risk ratio |
| SAE | serious adverse event |
| SAP | statistical analysis plan |
| SBP | systolic blood pressure |
| sd | standard deviation |
| U | unit |
| Umb art | umbilical arterial |
| yrs | years |
| µmol | micromole |

# Introduction

This document details the proposed presentation and analyses for the main analysis of short-term outcomes from the National Institute for Health Research (NIHR) Health Technology Assessment (HTA) programme funded multicentre randomised controlled trial PHOENIX. The results reported in this publication will follow the strategy set out here. The analysis of 6 and 24 months outcomes will be detailed in a separate statistical analysis plan. Subsequent analyses of a more exploratory nature will not be bound by this strategy, although they are expected to follow the broad principles described. The principles are not intended to curtail exploratory analysis (for example, to decide cut-points for categorisation of continuous variables), nor to prohibit accepted practices (for example, data transformation prior to analysis), but they are intended to establish the rules that will be followed, as closely as possible, when analysing and reporting the trial.

The analysis plan will be available on request when the principal manuscripts are submitted for publication. Suggestions for subsequent analyses by journal editors or referees will be considered carefully and carried out, as far as possible, in line with the principles of this analysis plan.

Any deviations from the statistical analysis plan will be described and the rationale given in the final report of the trial. The analysis will be carried out by an identified, appropriately qualified and experienced statistician, who will ensure the integrity of the data during processing. Examples of such procedures include quality control and evaluation procedures. This document and the interim and final analyses will be produced in line with NPEU Standard Operating Procedures ST 105 Statistical Analysis Plan; ST107 Statistical Analysis and Reporting.

**Chief Investigators**

Professor Andrew Shennan and Professor Lucy Chappell

Women’s Health Academic Centre, King’s College London

[andrew.shennan@kcl.ac.uk](mailto:andrew.shennan@kcl.ac.uk) and lucy.chappell@kcl.ac.uk

**Trial Statisticians**

Pollyanna Hardy and Melanie Greenland

[pollyanna.hardy@npeu.ox.ac.uk](mailto:pollyanna.hardy@npeu.ox.ac.uk) and [melanie.greenland@npeu.ox.ac.uk](mailto:melanie.greenland@npeu.ox.ac.uk) (to May 2017)

Louise Linsell and Virginia Chiocchia

[Louise.linsell@npeu.ox.ac.uk](mailto:Louise.linsell@npeu.ox.ac.uk) and [virginia.chiocchia@npeu.ox.ac.uk](mailto:virginia.chiocchia@npeu.ox.ac.uk) (from May 2017)

NPEU Clinical Trials Unit, University of Oxford

**Clinical Trials Unit Director**

Ed Juszczak

NPEU Clinical Trials Unit, University of Oxford

[ed.juszczak@npeu.ox.ac.uk](mailto:ed.juszczak@npeu.ox.ac.uk)

**Trial Co-ordinator**

Anna Placzek

NPEU Clinical Trials Unit, University of Oxford

anna.placzek@npeu.ox.ac.uk

# Background

PHOENIX is a multi-centre randomised controlled trial (RCT) to determine if planned early delivery for women with pre-eclampsia between 34^+0^ and 36^+6^ weeks of gestation reduces maternal adverse outcomes without substantial worsening of neonatal/infant outcomes, compared with the current practice of expectant management and delivery at 37 weeks of gestation.

The trial will recruit 900 women from approximately 25 consultant-led maternity units across England and Wales over three years.

# Description of primary and secondary outcomes

## Primary outcome

The primary outcomes are separated by short-term maternal outcome, short-term perinatal outcome, and long-term infant outcome. All short-term outcomes are collected from trial randomisation to discharge home.

**Primary short-term maternal outcome**

Composite of maternal morbidity of fullPIERS outcomes with the addition of recorded systolic blood pressure >=160 mmHg (with or without medication).

**Primary short-term perinatal outcome**

Composite of perinatal deaths (antenatal/intrapartum stillbirths and deaths within 7 days of delivery but not deaths due to congenital anomalies) or NNU admissions to infant hospital discharge.

**Primary long-term infant outcome (for reference)**

Neurodevelopmental assessment at 2 years of age corrected for prematurity using PARCA-R Parent Report Composite score.

## Secondary outcomes

The secondary outcomes are separated by short-term maternal outcomes, short-term perinatal outcomes, and long-term maternal outcomes.

**Secondary short-term maternal outcomes**

Tested:

- Composite of maternal morbidity components of fullPIERS outcomes
- Systolic blood pressure ≥160 mmHg (with or without medication)
- use of anti-hypertensive drugs,
- progression to severe pre-eclampsia (defined as systolic blood pressure >=160 mmHg, platelet count <100 x 10^9^/litre, abnormal liver enzymes (ALT or AST >70 iu/litre)),
- time and mode of onset (spontaneous, induced or pre-labour caesarean section) and mode of delivery (spontaneous vaginal delivery, assisted vaginal delivery, caesarean section),
- confirmed thromboembolic disease requiring anticoagulation,
- confirmed sepsis (positive blood or urine cultures),
- placental abruption

Not tested:

- estimated fetal weight (on ultrasound scan) <10^th^ centile,
- absent or reversed end diastolic flow (on umbilical artery Doppler),
- primary and additional indications for delivery in expectant management arm (maternal hypertension not controlled by maximal therapy, biochemical abnormality, haematological abnormality, fetal compromise on ultrasound scan, fetal compromise on cardiotocography, severe maternal symptoms, 37 weeks’ gestation or specified other)

**Secondary short-term perinatal outcomes**

Tested:

- antenatal/intrapartum stillbirths,
- neonatal death not related to congenital anomaly within 7 days of delivery
- NNU admission
- number of nights in each category of care (intensive, high dependency, special, transitional and normal), total number of nights in hospital,
- birth weight (g),
- customised/population birth weight centile (GROW),
- birth weight <10^th^ and <3^rd^ customised/population centile,
- gestational age at delivery,
- APGAR score at 5 minutes post-birth,
- need for supplementary oxygen prior to discharge,
- umbilical arterial and venous pH (and base excess) at birth,

Not tested:

- neonatal death prior to hospital discharge,
- number of days when supplemental oxygen is required,
- need for ventilation support (CPAP/high flow/endotracheal ventilation),
- pneumothorax (confirmed on chest X-ray),
- abnormal cerebral ultrasound scan,
- confirmed sepsis (positive blood or cerebrospinal fluid cultures),
- necrotising enterocolitis (Bell’s stage 2 and 3),
- seizures (confirmed by EEG or requiring anticonvulsant therapy),
- encephalopathy grade (worst at any time: mild, moderate, severe),
- hypoglycaemia (blood glucose <2.6 mmol/l on two or more occasions),
- other indications and main diagnoses resulting in NNU admission,
- exclusively breast-fed at discharge from the neonatal unit.

**Secondary long-term maternal outcomes (assessed at six months and two years of age corrected for prematurity) (for reference)**

Maternal physical and mental health using validated SF-12 questionnaire.

# Sample size and power

The sample size for the PHOENIX study is calculated on the ability to observe a clinically significant risk reduction in the primary short term maternal composite outcome of maternal morbidity and recorded systolic blood pressure of ≥160 mmHg, measured after randomisation.

**Superiority hypothesis in maternal outcome**

Based on data from the PELICAN study, 49 of 115 women with suspected pre-eclampsia (42.6%, 95% CI 33.4% to 52.2%) enrolled between 34^+0^ and 36^+6^ weeks of gestation developed maternal morbidity and hypertension of >=160 mmHg. Therefore, assuming an expected adverse maternal outcome incidence of 43% in the control group (expectant management), a sample size of 850 women will be needed to demonstrate a relative risk reduction of 25% to 32.25% (deemed clinically significant) with a 2-sided 5% significance level in the planned delivery group. Taking into account a 5% loss of women in follow-up, the overall target sample size for the study is 900 women (450 per group).

**Non-inferiority hypothesis in neonatal outcome**

A sample size of 850 women will result in approximately 860 live births (assuming 1 in 80 pregnancies are twin pregnancies). The PELICAN study2 reported that a composite of perinatal death or any neonatal admission occurred in 27 of 115 infants (23.5%; 95% CI 16.1% to 32.3%). Assuming a composite adverse neonatal outcome incidence of 24% in the control group (expectant management), a sample size of 860 (430 per group) will achieve 93% power to detect a non-inferiority margin of difference in incidence of no less than 10% and 78% power to detect a margin of no less than 8%.

In order to examine the component of perinatal death specifically, using Office for National Statistics (ONS) data for all babies born in England and Wales in 2013, of all pre-term births, 1.6% (585/36939) were perinatal deaths (stillbirth and early neonatal). A similar incidence is expected in women with pre-eclampsia as those deaths prevented by increased surveillance would be offset by pre-eclampsia associated co-morbidities of fetal growth restriction and placenta abruption. Thus, for the component of perinatal death, assuming a control group incidence of 1.5%, a sample size of 430 in each group would achieve 90% power to detect a non-inferiority margin of difference in incidence of no less than 2.7%. A non-inferiority margin of difference in incidence of no less than 2.3% would be detected with 79% power. If the control group incidence was 1%, then a margin of no less than 2.2% could be detected with 90% power, and 1.9% with 80% power.

For the component of neonatal unit admission, assuming a control group incidence of 21%, a sample size of 430 in each group will achieve 90% power to detect a non-inferiority margin of difference in incidence of no less than 9%. A non-inferiority margin of difference in incidence of no less than 8% would be detected with 82% power.

Assuming a loss to follow-up at two years of 20% we should obtain long term outcomes for approximately 690 infants (345 per group assuming no difference in the loss to follow-up between the groups). The PARCA-R questionnaire provides a composite score for neurodevelopment with a standardised mean of 100 and standard deviation of 15. With a one-sided significance level of 2.5%, under a non-inferiority hypothesis, a sample size of 345 in each group achieves a 94% power to detect a non-inferiority margin of difference in the mean PARCA-R score of no less than 4 points (1/4 of a standard deviation). A margin of no less than 3 points can be detected with 75% power.

# Random allocation

Randomisation will ensure balance on important prognostic factors using a minimisation algorithm to include the following factors: collaborating hospital, singleton or twin pregnancies, severity of hypertension in 48 hours prior to enrolment (highest systolic blood pressure with or without medication: <=149 mmHg, 150-159 mmHg, >=160 mmHg), parity, previous caesarean section and gestational age at randomisation (34/35/36 weeks).

# Protocol non-compliances

All protocol non-compliances will be listed in the final report. Non-compliances are defined below.

## Major

The following will be defined as major protocol non-compliances:

- Data considered fraudulent

## Minor

The following will be defined as minor protocol non-compliances:

**Participants randomised in error**

These include women:

- who are not between 34^+0^ and 36^+6^ weeks’ gestation inclusive
- who do not have pre-eclampsia or superimposed pre-eclampsia as defined in the inclusion criteria
- who do not have a singleton or diamniotic twin pregnancy
- who do not have a viable fetus
- who are under 18 years at time of randomisation
- whose consent to take part has not been fully documented
- for whom a decision has already been made to deliver within the next 48 hours

**Participants who do not receive allocated intervention**

These include women:

- in the ‘expectant management’ arm who received non-indicated delivery prior to 37 weeks’ gestation
- in the ‘planned immediate delivery’ arm who discontinued the intervention i.e. changed their mind after being randomised to ‘planned immediate delivery’ arm
- who were randomised to ‘planned immediate delivery’ but initiation of delivery is beyond 48 hours post-randomisation.

# Interim analyses

An independent Data Monitoring Committee (DMC) has been established, whose remit will be to review the trial’s progress. The DMC is independent of the trial organisers. Interim analyses will be supplied, in strict confidence, to the DMC, as frequently as the Chair requests. The terms of reference for the DMC were agreed at their first meeting and the DMC charter was finalised. Meetings of the committee will be arranged periodically, as considered appropriate by the Chair.

# Data collection schedule

## Before discharge

All data for this trial are routinely recorded clinical items that can be obtained from the clinical notes or local laboratory records. Clinical information will be collected using the following case report forms (CRFs):

1. Screening log
2. Eligibility
3. Maternal details
4. Prior to randomisation
5. EQ-5D
6. Contact details
7. Abnormal lab parameters
8. Delivery
9. Maternal discharge
10. Maternal outcomes
11. Infant delivery
12. Infant discharge
13. Incident report form
14. Withdrawal form
15. SAE form

# Derivation of variables

See associated data derivation document.

# Participant groups for analysis

## Primary analysis strategies

For all short-term maternal outcomes, the primary inference will be based on an intention to treat (ITT) analysis, i.e. women will be analysed in the groups into which they were randomly allocated, regardless of allocation received.

For all short-term perinatal outcomes, both an ITT and per protocol analysis (PP) will be performed and inference will be based on both.

The unit of analysis for all maternal outcomes is the woman. Women can be randomised into the trial for a subsequent pregnancy during the trial period. We expect this to occur for less than 1% of the women recruited. The number of times this occurs will be reported and they will be treated as independent observations when describing the trial population. However, this raises the issue of non-independence of observations for maternal outcomes and perinatal outcomes between siblings. These correlations will be accounted for in the analysis of all maternal and perinatal outcomes (see section 12).

The unit of analysis for all perinatal outcomes is the baby. This raises the issue of non-independence of observations if the mother gives birth to twins. It is expected that the rate of twin births will be between 1% and 5%. In addition to accounting for the correlation in outcomes between siblings of women randomised twice, the correlation in outcomes between twins will also be accounted for in the analysis of all perinatal outcomes (see section 12).

Firstly an unadjusted analysis will be performed followed by an adjusted analysis by the minimisation factors used at randomisation where possible: hospital, singleton/twin pregnancies, severity of hypertension in 48 hours prior to enrolment, parity, previous caesarean section and gestational age at randomisation. The primary inference for both the ITT and PP populations will be based on the adjusted analysis.

## Post randomisation exclusions

Exclusions to the analysis population post randomisation consist of the following:-

- Women for whom a consent form was not received
- Women for whom consent to use their data was withdrawn

(Women can specify whether data collected up to the point of withdrawal can be used. If the response is ‘No’, then they will be considered post-randomisation exclusions. If the response is ‘Yes’, then they will be reported as ‘missing’ for any data not collected after withdrawal).

- Women for whom an entire record of fraudulent data was detected

(Should fraudulent data be detected, consideration will be given to excluding all data for the site where such data was found).

The numbers (with percentages of the randomised population) of post-randomisation exclusions will be reported by randomised treatment group, and reasons summarised.

## Descriptive analysis population

Baseline demographic and clinical characteristics will be reported for all women randomised excluding post-randomisation exclusions (see section 10.2).

## Comparative analysis population

**Short-term maternal outcomes**

All women randomised minus post-randomisation exclusions (see section 10.2).

**Short-term perinatal outcomes**

Since the hypothesis being tested for these outcomes is a non-inferiority hypothesis, both an ITT and PP will be undertaken.

The PP population will include the babies of all mothers randomised, minus post-randomisation exclusions (see section 10.2), minus those randomised in error (see section 6.2) and minus those who did not receive the allocated intervention (see section 6.2).

Evidence suggesting non-inferiority will be concluded if results using both populations are consistent with each other.

## Interim analysis population

Different denominators will be used in the interim analysis: baseline data will be reported for all women with available data, excluding known post-randomisation exclusions (see section 10.2). Outcome data will be reported for all women and babies of women who have delivered and been discharged home at the time of the database snapshot, excluding known post-randomisation exclusions (see section 10.2). The short-term primary perinatal outcome will be reported for both the ITT and the PP population.

# Descriptive analyses

## Representativeness of trial population and participant throughput

The flow of participants through each stage of the trial will be summarised by randomised group using a CONSORT diagram. This will describe the following numbers of women:

- Assessed for eligibility
- Non-eligible
- Eligible
- Declined
- Total randomised
- Allocated to expectant management
- Allocated to planned immediate delivery
- Included in the analysis population for the short-term co-primary outcomes

## Baseline comparability of randomised groups

Women in the two randomised groups will be described separately with respect to maternal characteristics at trial entry.

**Maternal characteristics**

The following maternal characteristics will be described for the women entered into the study:

- Gestational age at diagnosis (weeks)
- Highest BP reading that lead to diagnosis
- Diagnosis of pre-eclampsia
- Gestational age at randomisation
- Pregnancy type
- Number of live fetuses at study entry
- Comorbidity at study entry
- Highest BP reading in 48 hours prior to study entry
- Antihypertensive medication at study entry
- Aspirin taken during pregnancy
- Gestational age aspirin first prescribed
- LMWH received during pregnancy
- Gestational age LMWH first prescribed
- Most recent proteinuria reading
- Most recent lab parameters prior to study entry
- Assessment of the cervix
- Bishop score at study entry
- Fetal growth scan completed in the last two weeks
- Suspected fetal growth restriction at trial entry
- In-patient at time of trial entry

The number and percentage will be presented for binary and categorical variables. The mean and standard deviation or the median and the interquartile range will be presented for continuous variables, or the range if appropriate. There will be no tests of statistical significance performed nor confidence intervals calculated for differences between randomised groups on any baseline variable.

## Loss to follow-up

The number and percentage of losses to follow up among women will be reported for the two trial arms, and the reasons will be recorded. All deaths of the woman or baby will be reported separately.

## Description of adherence to intervention

A summary of adherence to the intervention allocated will be reported. This will include summaries of time between randomisation and delivery, and time between randomisation and initiation of delivery, overall and by gestational age group (34^+0^ to 34^+6^ weeks, 35^+0^ to 35^+6^ weeks and 36^+0^ to 36^+6^ weeks). Indication leading to decision to deliver will also be reported.

# Comparative analysis

Demographic factors and clinical characteristics will be summarised with counts and percentages for categorical variables, means and standard deviations for normally distributed continuous variables, or medians and interquartile ranges for other non-normally distributed continuous variables.

Women and babies of women will be analysed by ITT or PP inference as described in section 10.4. The expectant management group will be used as the reference group in all analyses.

For binary outcomes unadjusted and adjusted relative risks and confidence intervals will be estimated using a log binomial regression model, or using a log Poisson regression model with a robust variance estimator if the binomial model fails to converge. Analyses will be adjusted for all minimisation factors where possible (i.e. collaborating hospital, singleton/twin pregnancies, severity of hypertension in 48 hours prior to enrolment, parity, previous caesarean section and gestational age at randomisation). Collaborating hospital will be treated as a random effect in the model, and all other factors as fixed effects. For continuous outcomes unadjusted and adjusted mean differences and confidence intervals will be estimated using linear regression assuming residuals are normally distributed. Should this assumption be considered unmet, quantile regression methods will be used.

Where possible, analyses (both unadjusted and adjusted) will also account for the correlation in outcomes for women and siblings born in a subsequent pregnancy during the trial period and for the correlation in outcomes between twins by treating these as random effects in the model.

Analysis of secondary outcomes will be clearly delineated from that of the primary outcomes in any statistical reports produced.

# Pre-specified subgroup analysis

Pre-specified subgroup analyses for the short-term co-primary outcomes will be undertaken for parity (0 and ≥1 previous pregnancy), mild versus all other degrees of hypertension, gestation at the time of randomisation (34/35/36 weeks) and for singleton versus twin pregnancy. This will be conducted on the ITT population for maternal outcome and by ITT and PP analysis for the perinatal outcome. The consistency of the effect of planned delivery versus expected management across subgroups will be assessed using the standard statistical test of interaction, in addition to the adjusted model. Results will be presented on forest plots with risk ratios, 95% confidence intervals and the results of the interaction test.

# Sensitivity analysis

The analyses for the short-term primary maternal and perinatal outcomes will be repeated excluding women (and their babies) in the planned delivery arm whose initiation of delivery was delayed. For this sensitivity analysis, this threshold will be set at >96 hours post-randomisation to allow for clinical (e.g. steroid administration) and logistical (e.g. availability of labour ward bed or neonatal unit cot) delays. The analysis populations will be ITT for the primary maternal outcome and PP (with the more relaxed criteria of delays >96 hours post-randomisation) for the primary perinatal outcome.

If an entire record is considered fraudulent, this will be excluded from all analyses (see section 10.2). However, if any individual data is considered fraudulent, a sensitivity analysis will be conducted on outcomes where any fraudulent data is detected excluding data considered fraudulent.

# Safety data analysis

Serious adverse events will be listed by allocation.

# Statistical significance

For all analyses, a 95% confidence interval will be calculated.

# Procedure for accounting for missing data

Missing data will be described, for example, by presenting the number and percentage of individuals in the missing category. All data collected on data collection forms will be used, since only essential data items will be collected.

# Procedure for reporting deviation(s) from the original statistical plan

Deviations from the original statistical plan are unlikely, however should they occur, they will be documented in the final analysis report.

# Statistical software employed

The statistical software Stata/SE version 15 (or later) for Windows will be used for all analyses.

# Additional exploratory analysis

Any analyses not specified in the analysis protocol will be exploratory in nature and a two-sided significance level of 0.01 will be used with 99% confidence intervals. All such analyses will be approved by the Co-investigator Group.

# Dummy tables

Dummy tables are displayed in the ‘Dummy tables’ word document.

# References

Should multiple imputation be the method of choice for handling missing data in randomised trials? Sullivan TR, White IR, Salter AB, Ryan P, Lee KJ. Statistical methods in medical research (2016) doi: 10.1177/0962280216683570.

# Approvals

| **TSC Chair** | | | |
| --- | --- | --- | --- |
| **Print Name:** | Professor Jane Norman | **Signature:** |  |
| **Job Title:** | Professor of Maternal and Fetal Health, University of Edinburgh | **Date:** |  |
| **Chief Investigators** | | | |
| **Print Name:** | Professor Andrew Shennan | **Signature:** |  |
| **Job Title:** | Women’s Health Academic Centre,  St Thomas’ Hospital | **Date:** |  |
| **Print Name:** | Professor Lucy Chappell | **Signature:** |  |
| **Job Title:** | Women’s Health Academic Centre,  St Thomas’ Hospital | **Date:** |  |
| **Senior Trial Statistician** | | | |
| **Print Name:** | Louise Linsell | **Signature:** |  |
| **Job Title:** | Senior Statistician,  NPEU Clinical Trials Unit,  University of Oxford | **Date:** |  |
